# Diagnostic value of interferon-γ release assay in patients with COPD complicated with pulmonary tuberculosis

**DOI:** 10.1186/s12879-025-10523-3

**Published:** 2025-01-22

**Authors:** Yanxiao Rong, Haibin Wang, Yuanyuan Su, Qian Wang, Xuepei Sun, Wei Wang

**Affiliations:** 1 The First Department of Tuberculosis, Fifth Hospital of Shijiazhuang, Shijiazhuang, Hebei China; 2 The Third Department of Infection, Fifth Hospital of Shijiazhuang, Shijiazhuang, Hebei China

**Keywords:** COPD, Pulmonary tuberculosis, Interferon-γ release assay

## Abstract

**Background:**

The common diagnostic methods for tuberculosis have been showing reduced sensitivity among chronic obstructive pulmonary disease patients. This study was conducted to evaluate and analyse the diagnostic value of an interferon-γ release assay in COPD patients complicated with pulmonary tuberculosis.

**Methods:**

A nested case-control study was conducted on 123 COPD patients hospitalized at the Fifth Hospital of Shijiazhuang, Hebei Province, from January 2019 to June 2021. Thirty-one patients with active pulmonary tuberculosis complicated with COPD composed the observation group (Group A), 31 patients with nonactive pulmonary tuberculosis complicated with COPD composed the COPD control group (Group B), and 31 patients with active pulmonary tuberculosis not complicated with COPD composed the non-COPD control group (Group C). An interferon-γ release assay, a purified protein derivative of tuberculin (PPD) test, an anti-tuberculosis antibody test, a test of *Mycobacterium tuberculosis by* sputum smear microscopy and a test of *Mycobacterium tuberculosis* by PCR method were used to test patients in each group. The positive detection rates generated from the five test methods were compared and analysed.

**Results:**

In COPD patients complicated with active pulmonary tuberculosis, the differences in the percentage of patients with positive interferon-γ release between the PPD test, *anti-tuberculosis antibody test*, *Mycobacterium tuberculosis* by sputum smear microscopy and PCR test results were statistically significant.

**Conclusion:**

In patients with COPD complicated with active pulmonary tuberculosis, the percentage of patients who were positive according to the interferon-γ release assay was higher than that according to the sputum smear microscopy, PCR detection of *Mycobacterium tuberculosis* in sputum specimen, and detection of anti-tuberculosis antibodies. COPD-related complications did not affect the T-SPOT; the greater the T-SPOT value was, the greater the likelihood of active TB. For patients who are T-SPOT positive but clinically considered to have inactive tuberculosis, regular follow-ups should be performed to observe changes in the patient’s condition.

## Background

Chronic obstructive pulmonary disease (COPD) is a common and frequently occurring respiratory disease and a major chronic lung disease that seriously threatens public health and working capacity [1, 2]. Among people over 40 years old in China, the prevalence of COPD is as high as 8.2% [3, 4]. Long-term and repeated respiratory infections may lead to a reduction in local respiratory immunity in COPD patients. Moreover, the use of systemic or inhaled corticosteroids can also reduce the incidence of autoimmunity in COPD patients and increase the probability of developing infection or tuberculosis [5]. The decreased expression rates [6, 7] of NK cells and CD4 + T lymphocytes and decreased cellular immune function in patients with COPD complicated with pulmonary tuberculosis may lead to false negative detection results in serological and PPD skin test results, resulting in a decrease in the sensitivity of these two detection methods [8, 9]. Therefore, finding a sensitive detection method has important clinical value and social significance, particularly for diagnosing pulmonary tuberculosis in COPD patients.

Pulmonary tuberculosis is a chronic infectious disease that seriously endangers the health of patients. It is a public health and social problem of global focus [10]. Although substantial achievements have been made in the field of tuberculosis prevention and control, tuberculosis is still one of the most serious public health threats globally [11]. On October 28, 2015, the World Health Organization released the 2015 Global Tuberculosis Report. The latest statistics show that there were 9.6 million new cases of tuberculosis worldwide in 2014, of which 1.5 million people died of tuberculosis [12]. According to WHO estimates, the number of new cases of pulmonary tuberculosis in China in 2014 was 930,000 [13], ranking third in the world after India (2.2 million) and Indonesia (1 million). Although the number of new cases in 22 countries with a high burden of tuberculosis worldwide has decreased, the situation is still not optimistic [14]. The results of a national epidemiological survey of tuberculosis in 2010 showed that the total prevalence rates of active, smear-positive and anti-*mycobacterium tuberculosis* antibody-positive pulmonary tuberculosis in China were 459/100,000, 66/100,000 and 119/100,000, respectively [15]. The total registered positive rates of patients aged 40–80 in the Fifth Hospital of Shijiazhuang were 791/100,000. The incidence of pulmonary tuberculosis gradually increased with age (the highest incidence rate of pulmonary tuberculosis was in the group aged ≥ 60 years). The prevalence rates of active pulmonary tuberculosis and anti-*mycobacterium tuberculosis* antibody-positive pulmonary tuberculosis among rural populations were both significantly greater than those among urban populations. The differences in the prevalence of pulmonary tuberculosis in the eastern, central and western regions were significant [15].

With all this information, it is evident that patients with COPD have a higher risk of developing pulmonary tuberculosis due to their immunosuppressive condition and the frequent use of corticosteroids. The common diagnostic methods for tuberculosis have been showing reduced sensitivity among COPD patients. This highlights the need for diagnostic tests that are widely accessible, have higher acceptability, are easy to perform and, most importantly, possess higher sensitivity in detecting tuberculosis. Therefore, we designed this study to analyse the diagnostic sensitivity of the interferon-γ release assay in the COPD population complicated with pulmonary tuberculosis.

## Methods

### Subjects and design

Data from patients admitted to the Respiratory and Critical Care Department of the Fifth Hospital of Shijiazhuang in Hebei Province from September 2013 to September 2015 were retrospectively analysed. The inclusion criteria for patients were as follows: (1) aged 40–90 years, regardless of sex; (2) diagnosed with active or previous pulmonary tuberculosis according to the Guidelines for Diagnosis and Treatment of Pulmonary Tuberculosis issued by the Chinese Medical Association Tuberculosis Branch [16]; and (3) diagnosed with chronic obstructive pulmonary disease (COPD) according to the Global Initiative for Chronic Obstructive Pulmonary Disease (GOLD) Guidelines developed by the WHO [17]. The exclusion criteria were as follows:1) patients complicated with HIV infection; 2) patients complicated with diabetes or abnormal blood glucose; 3) patients who used immunosuppressants; and (4) patients complicated with rheumatic and immune-related diseases. All patients who met al. l the inclusion criteria and did not meet any exclusion criteria were included in a nested case-control study [18]. Thirty-one patients with active pulmonary tuberculosis complicated with COPD years were selected as the observation group (Group A), 31 patients with nonactive pulmonary tuberculosis complicated with COPD were selected as the COPD control group (Group B), and 31 patients with active pulmonary tuberculosis not complicated with COPD were selected as the non-COPD control group (Group C).

### Test methods

#### Blood mixed lymphocyte culture + interferon-γ (T-SPOT) assay

In the T-SPOT test, tuberculosis-specific antigen (ESAT-6/CFP 10) was used based on the enzyme-linked immunospot assay (ELISPOT) to detect the presence of tuberculosis effector T lymphocytes in the subjects to determine whether the subjects were currently infected with *Mycobacterium tuberculosis*. The enzyme-linked immunospot technology (ELISPOT) principle involves capturing the cytokines secreted by cultured cells with antibodies and presenting the cytokines using the enzyme-linked immunosorbent spot (ELISPOT) assay method. Tuberculosis infection T-cell detection (T-SPOT. TB) kit for *Mycobacterium tuberculosis* infection (Oxford Immunotec, UK) was used. The test kit contained two types of tuberculosis-specific antigens: *M. tuberculosis*-specific mixed peptide A, mainly composed of the ESAT-6 antigen, and M. tuberculosis-specific mixed peptide B, mainly composed of the CFP10 antigen. The specific steps were performed by strictly following the instructions. Result determination: The results of both the T-SPOT A and T-SPOT B were considered positive if the number of spots was ≥ 24.

#### PPD skin test

0.1 ml of PPD (5 IU) was pipetted, and the area at the junction between the middle one-third and half of the forearm at the palm side was used for the intradermal injection. The injection site reaction was checked 72 h after injection. The result was judged as positive if the average of the maximum and the minimum diameters of the induration was ≥ 10 mm.

#### Serum *anti-mycobacterium tuberculosis* antibody detection

A *Mycobacterium tuberculosis*-specific antibody detection kit (MP Biomedicals Asia Pacific Pte Ltd. in Singapore) was used for analysis, and the specific testing procedures and results were determined by strictly following the instructions for use.

#### *Mycobacterium tuberculosis* sputum smear microscopy

After admission, the morning sputum specimens were collected from the patients for three consecutive days (care was taken not to mix it with saliva), and smear microscopy was conducted by experienced laboratory physicians in the microbiology department of our hospital. Polymerase chain reaction (PCR) detection of *Mycobacterium tuberculosis* in sputum specimens: On the next day after admission, the morning sputum specimens were collected from the patients (care was taken not to mix it with saliva) and tested using the *Mycobacterium tuberculosis* nucleic acid amplification (PCR) fluorescence detection kit (Daan Gene Co., Ltd. of Sun Yat-Sen University). The specific steps and judgement methods were performed by strictly following the instructions for use.

### Statistical processing

The data were cleaned and analyzed using SPSS 18.0 statistical software. The continuous data were presented as mean ± standard deviation and median. The categorical data were presented as frequencies and percentages. The Pearson chi-square (χ^2^) test was used to compare the positive rates of each group. The Mann-Whitney U test was used if the intergroup mean values did not follow a normal distribution. *P* < 0.05 was considered to indicate statistical significance.

## Results

### Characteristics of the study population

A total of 93 participants were divided equally into three groups (Group A, Group B, and Group C). The mean age of the total participants was 65.1 years, with a standard deviation of 10.6 years, and the median age was 66 years. Most participants in Group A were aged 56–70 (61.2%), while Groups B and C had a higher representation in the 71– 90 years age range (48.4% and 38.7%, respectively). The participants of Groups A, B, and C had a mean, standard deviation, and median of 62.7 ± 9.2 (64), 67.7 ± 11.6 (70), and 64.8 ± 10.7 (65), respectively. Group A and Group B predominantly consisted of males (87.1%), while C had a balanced sex distribution, with 45.2% males vs. 54.8% females. The average number of hospitalizations across all groups was 1.22 ± 0.56, with the majority experiencing only one hospitalization. The average length of hospitalization was 9.5 ± 4.1 days. Most patients from all three groups had to stay in the hospital for 8–14 days. Among the participants of all groups, pulmonary tuberculosis was the most frequent primary diagnosis in Groups A (78.6%) and C (71.0%). In contrast, in Group B, most of the patients had been diagnosed with different variants of COPD (85.2%). Other less common diagnoses included pneumonia, tuberculous pleurisy, and bronchiectasis with infection (Table 1).

**Table 1:** Demographic and clinical characteristics of the study participants

Variables	Group A *n* (Col %)	Group B *n* (Col %)	Group C *n* (Col %)	Mean ± SD, (Median)
**Age (in years)**				**65.1 ± 10.6, (66)**
40–55	6 (19.4%)	6 (19.4%)	7 (22.6%)	
56–70	19 (61.2%)	10 (32.2%)	12 (38.7%)	
71–90	6 (19.4%)	15 (48.4%)	12 (38.7%)	
**Sex**				
Male	27 (87.1%)	21 (67.7%)	14 (45.2%)	
Female	4 (12.9%)	10 (32.3%)	17 (54.8%)	
**Number of Hospitalization**, *n* = 72				**1.22 ± 0.56**,** (1)**
1	9 (64.3%)	22 (81.5%)	29 (93.6%)	
2	4 (28.6%)	4 (14.8%)	1 (3.2%)	
3 to 4	1 (7.1%)	1 (3.7%)	1 (3.2%)	
**Length of hospitalization (in days)**, *n* = 72				**9.5 ± 4.1**,** (9)**
At least 3	1 (7.1%)	0 (0)	1 (3.2%)	
4–7	3 (21.4%)	4 (14.8%)	13 (41.9%)	
8–14	8 (57.1%)	17 (63.0%)	16 (51.6%)	
> 14	2 (14.3%)	6 (22.2%)	1 (32.0%)	
**Primary Diagnosis**, *n* = 72				
Acute COPD	0 (0)	16 (59.3%)	0 (0)	
Bronchiectasis with Infection	0 (0)	0 (0)	1 (3.2%)	
COPD	0 (0)	3 (11.1%)	0 (0)	
COPD with Acute Lower RTI	0 (0)	4 (14.8%)	0 (0)	
Infiltrative Pulmonary Tuberculosis	0 (0)	0 (0)	1 (3.2%)	
Interstitial Pneumonitis	0 (0)	0 (0)	1 (3.2%)	
Lung Shadow	2 (14.3%)	0 (0)	2 (6.5%)	
Pleural Effusion	0 (0)	1 (3.7%)	0 (0)	
Pneumonia	1 (7.1%)	2 (7.4%)	1 (3.2%)	
Pulmonary Tuberculosis	11 (78.6%)	0 (0)	22 (71.0%)	
Secondary Pulmonary Arterial Hypertension	0 (0)	1 (3.7%)	0 (0)	
Tuberculous Pleurisy	0 (0)	0 (0)	3 (9.7%)	

### Observation of positive detection rates of 5 diagnostic methods in group A and group B

2.2.1 The comparison results of the positive detection rate of the T-SPOT test versus that of the PPD skin test, Serum *anti-mycobacterium tuberculosis* antibody detection test, *Mycobacterium* tuberculosis sputum smear microscopy, and PCR detection of *Mycobacterium tuberculosis* in sputum specimen in Group A are shown in Table 2.

**Table 2:** Comparison of positive detection rates by T-SPOT and PPD skin test, serum *anti-mycobacterium tuberculosis* antibody detection test, *Mycobacterium* tuberculosis Sputum smear microscopy, and PCR detection of *Mycobacterium tuberculosis* in sputum specimen for patients in Group A

Test Method	Positive (%)	Negative (%)	Total
T-SPOT A	24 (77.4%)	7 (22.6%)	31
T-SPOT B	24 (77.4%)	7 (22.6%)	31
PPD skin test	21 (67.7%)	10 (32.3%)	31
Serum *anti-mycobacterium tuberculosis* antibody detection test	8 (25.8%)	23 (74.2%)	31
*Mycobacterium Tuberculosis* sputum smear microscopy	5 (16.1%)	26 (83.9%)	31
PCR detection of *mycobacterium tuberculosis* in sputum specimen	8 (25.8%)	23 (74.2%)	31

A pairwise comparison of the above results revealed that the difference in the positive detection rate of the T-SPOT test versus PPD was χ2 = 0.83, *P* > 0.05, with no statistically significant difference; the differences in the positive detection rates of the T-SPOT test versus anti-tuberculosis antibody detection and PCR of sputum specimens were χ2 = 16.53, *P* < 0.01, with a statistically significant difference; and the difference in the positive detection rate of the T-SPOT test versus sputum tuberculosis smear microscopy was χ2 = 23.39, *P* < 0.01, with a statistically significant difference.

2.2.2 Comparison of the rates of positive T-SPOT test and PPD skin test, Serum *anti-mycobacterium tuberculosis* antibody detection test, *Mycobacterium tuberculosis* sputum smear microscopy, and PCR detection of *Mycobacterium tuberculosis* in sputum specimen results in Group B are shown in Table 3.

**Table 3:** Comparison of positive detection rates by T-SPOT test and PPD skin test, serum *anti-mycobacterium tuberculosis* antibody detection test, *Mycobacterium tuberculosis* Sputum smear microscopy, and PCR detection of *Mycobacterium tuberculosis* in sputum specimen results in Group B

Test Method	Positive (%)	Negative (%)	Total
T-SPOT A	20 (64.5%)	11 (35.5%)	31
T-SPOT B	19 (61.3%)	12 (38.7%)	31
PPD skin test	14 (45.2%)	17 (54.8%)	31
Serum *anti-mycobacterium tuberculosis* antibody detection test	9 (29.0%)	22 (71.0%)	31
*Mycobacterium tuberculosis* sputum smear microscopy	0 (0.0%)	31 (100.0%)	31
PCR detection of *mycobacterium tuberculosis* in sputum specimen	0 (0.0%)	31 (100.0%)	31

A pairwise comparison of the above results revealed that the difference in the positive detection rates of T-SPOT A versus PPD skin test was χ2 = 2.34, *P* > 0.05, with no statistically significant difference; the difference in the positive detection rate of T-SPOT B versus PPD skin test was χ2 = 1.62, *P* > 0.05, with no statistically significant difference; the difference in the positive detection rate of T-SPOT A versus Serum *anti-mycobacterium tuberculosis* antibody detection test was χ2 = 7.84, *P* < 0.05, with a statistically significant difference; the difference in the positive detection rates of T-SPOT B versus Serum *anti-mycobacterium tuberculosis* antibody detection test was χ2 = 6.51, *P* < 0.05, with a statistically significant difference; and the differences in the positive detection rate of T-SPOT versus *Mycobacterium tuberculosis* sputum smear microscopy and PCR detection of *Mycobacterium tuberculosis* in sputum specimen were χ2 = 26.1, *P* < 0.01, with a statistically significant difference.

2.2.3 Comparison of the rates of positive T-SPOT test and PPD skin test, Serum *anti-mycobacterium tuberculosis* antibody detection test, *Mycobacterium tuberculosis* sputum smear microscopy, and PCR detection of *Mycobacterium tuberculosis* in sputum specimen results in Group C are shown in Table 4.

**Table 4:** Comparison of positive detection rates by T-SPOT test and PPD skin test, serum *anti-mycobacterium tuberculosis* antibody detection test, *Mycobacterium tuberculosis* Sputum smear microscopy, and PCR detection of *Mycobacterium tuberculosis* in sputum specimen for patients in Group C

Test Methods	Positive (%)	Negative (%)	Total
T-SPOT A	27 (87.1%)	4 (12.9%)	31
T-SPOT B	27 (87.1%)	4 (12.9%)	31
PPD skin test	22 (71.0%)	9 (29.0%)	31
Serum *anti-mycobacterium tuberculosis* antibody detection test	9 (29.0%)	22 (71.0%)	31
*Mycobacterium tuberculosis* sputum smear microscopy	7 (22.6%)	24 (77.4%)	31
PCR detection of *mycobacterium tuberculosis* in sputum specimen	9 (29.0%)	22 (71.0%)	31

A pairwise comparison of the above results revealed that the difference in the positive detection rate of the T-SPOT test versus PPD skin test was χ2 = 2.43, *P* > 0.05, with no statistically significant difference; the differences in the positive detection rates of the T-SPOT test versus Serum *anti-mycobacterium tuberculosis* antibody detection test and PCR detection of *Mycobacterium tuberculosis* in sputum specimen were χ2 = 21.46, *P* < 0.01, with a statistically significant difference; and the difference in the positive detection rate of the T-SPOT test versus *Mycobacterium tuberculosis* sputum smear microscopy was χ2 = 26.1, *P* < 0.01, with a statistically significant difference.

### Intergroup comparison of positive detection rates between different test methods

2.3.1 Intergroup comparison of positive detection rates using different test methods between Group A and Group C is shown in Table 5.

**Table 5:** Intergroup Comparison of Positive Detection Rates using different test methods in Group A and Group C

Test Methods	Group A	Group C	χ^2^	*P*
T-SPOT A	24 (77.4%)	27 (87.1%)	0.99	*P* > 0.05,
T-SPOT B	24 (77.4%)	27 (87.1%)	0.99	*P*>0.05
PPD skin test	21 (67.7%)	22 (71.0%)	0.08	*P*>0.05
Serum *anti-mycobacterium tuberculosis* antibody detection test	8 (25.8%)	9 (29.0%)	0.08	*P*>0.05
*Mycobacterium tuberculosis* sputum smear microscopy	5 (16.1%)	7 (22.6%)	0.41	*P*>0.05
PCR detection of *mycobacterium tuberculosis* in sputum specimen	8 (25.8%)	9 (29.0%)	0.08	*P*>0.05

A comparison of the positive detection rates of different test methods between Group A and Group C revealed that the positive rates of Group A were slightly lower than those of Group C. The χ^2^ test was used to statistically analyse the positive detection rates of different detection methods between Group A and Group C. All the *P* values were > 0.05, with no significant difference.

2.3.2 Intergroup comparison of positive detection rates using different test methods between Group A and Group B is shown in Table 6.

**Table 6:** Intergroup Comparison of Positive Detection Rates Using Different Detection Methods in Group A and Group B

Test Method	Group A	Group B	χ^2^	*P*
T-SPOT A	24 (77.4%)	20 (64.5%)	1.25	*P*>0.05
T-SPOT B	24 (77.4%)	19 (61.3%)	2.47	*P*>0.05
PPD skin test	21 (67.7%)	8 (23.8%)	10.95	*P*<0.01
Serum *anti-mycobacterium tuberculosis* antibody detection test	8 (25.8%)	9 (29.0%)	1.18	*P*>0.05

The ${\chi^2}$ test was used to compare the T-SPOT-positive detection rates of Group A and Group B, and the *P* value was < 0.01, indicating a statistically significant difference.

## Intergroup comparisons of differences in T-SPOT test results mean values

### Intergroup comparisons of the differences in T-SPOT test resutls mean values between group A and group C

The T-SPOT A and T-SPOT B values of Group A and Group C did not follow a normal distribution; therefore, the Mann-Whitney U test was used. The mean T-SPOT A values of Group A and Group C were Z=-1.227 and *P* = 0.220, indicating no statistically significant difference between the two groups. The mean T-SPOT-A values of Group A and Group C were Z=-1.187 and *P* = 0.235, indicating no statistically significant difference between the two groups.

### The difference in the mean T-SPOT positive detection rate between Group A and Group B

The T-SPOT A and T-SPOT B values of Group A and Group B did not follow a normal distribution; therefore, the Mann-Whitney U test was used. The difference in the T-SPOT A values between Group A and Group B was Z=-1.454, *P* = 0.146, indicating no statistically significant difference between the two groups. The mean T-SPOT-A positive detection rate between Group A and Group B was Z=-2.313, *P* = 0.021, indicating a statistically significant difference between the two groups.

## Discussion

This study demonstrated that in patients with COPD complicated with active pulmonary tuberculosis, the diagnostic sensitivity of the interferon-γ release assay was higher than that of *Mycobacterium tuberculosis* sputum smear microscopy, PCR detection of *Mycobacterium tuberculosis* in sputum specimen, and Serum *anti-mycobacterium tuberculosis* antibody detection test. COPD-related complications did not affect the T-SPOT; the greater the T-SPOT value was, the greater the likelihood of active TB. For patients who are T-SPOT positive but clinically considered to have inactive tuberculosis, regular follow-ups should be performed to observe changes in the patient’s condition.

This study used an interferon-γ release assay (T-SPOT), a PPD skin test, *Mycobacterium tuberculosis* sputum smear microscopy, PCR detection of *Mycobacterium tuberculosis* in sputum specimens, and a Serum *anti-mycobacterium tuberculosis* antibody detection test to detect *Mycobacterium tuberculosis* in COPD patients complicated with tuberculosis, and the positive detection rates were analysed and compared between the groups. Tables 2 and 3 show that in groups A and B, the T-SPOT positivity rates were significantly greater than those of PPD skin test, Serum *anti-mycobacterium tuberculosis* antibody detection test, *Mycobacterium tuberculosis* sputum smear microscopy, and PCR detection of *Mycobacterium tuberculosis* in sputum specimen. Statistical analysis revealed no significant difference in the percentage of positive cells detected between the T-SPOT and PPD skin test methods. However, there was a significant difference in the rates between the T-SPOT and *Mycobacterium tuberculosis* sputum smear microscopy and PCR detection of *Mycobacterium tuberculosis* in sputum specimens test methods. However, the antigen contained in PPD skin test is a mixture of antigens of pathogenic *Mycobacterium tuberculosis*, environmental *Mycobacterium tuberculosis*, and BCG strains, and the results are easily affected by BCG vaccination and other environmental Mycobacterium infections, thus resulting in false positive results [19]. However, T-SPOT was used to detect activated T cells formed after stimulation with tuberculosis-specific antigens in anticoagulated human peripheral blood, and the *Mycobacterium tuberculosis*-specific antigens ESAT-6 and CFP-10 are missing in both BCG and environmental mycobacteria; therefore, T-SPOT test has high specificity [20] and greater diagnostic value in the diagnosis of tuberculosis infection than the PPD skin test. The above results may be related to the detection methods and sputum quality used [20]. Although etiological diagnosis is the gold standard for the diagnosis of tuberculosis [21], due to the influence of many factors, such as the location of the lesion, the nature of the lesion, the drainage bronchus, the quality of sputum, and the capacity of testing technology, the positive detection rate of PCR detection of *Mycobacterium tuberculosis* in sputum specimens was only 55.28%.

Aetiological diagnosis (such as the widely used NGS) and pathological diagnosis are the gold standards for detection, but in some general hospitals, etiological diagnosis is not available, and PPD skin tests are not possible. In addition, some patients cannot tolerate bronchoscopy, and sputum smears are not feasible. In addition, patients with COPD cannot tolerate the tests available for etiological and pathological diagnosis. Interferon-γ release assay (T-SPOT) is a non-invasive hematological test that is easy to perform and can be tolerated by patients. The test effectively distinguishes acute and chronic conditions, demonstrating its acceptability and feasibility [22]. Therefore, this test method is still of important reference value, especially for doctors in general hospitals.

In patients with COPD complicated with active pulmonary tuberculosis, the positive detection rate of the interferon-γ release assay is higher than that of *Mycobacterium tuberculosis* sputum smear microscopy, PCR detection of *Mycobacterium tuberculosis* in sputum specimens and Serum *anti-mycobacterium tuberculosis* antibody detection test. A recent systematic review also reported better pooled predictive ability of interferon-γ release assay compared to other methods of TB diagnosis [23]. Although some authors had found that the TSPOT test did not confidently assist in confirming the diagnosis of active TB, no study had entirely ruled out its usefulness [24].

Table 3 shows data of some patients with inactive pulmonary tuberculosis tested positive according to the T-SPOT. The reason is that for patients clinically diagnosed with inactive tuberculosis, there may be potential *Mycobacterium tuberculosis* in previous lesions, which secretes *Mycobacterium tuberculosis*-specific antigens to stimulate the formation of activated effector T cells, leading to T-SPOT positivity. Regular follow-up should be conducted in clinical practice to detect changes in the condition of patients in a timely manner.

Table 5 shows no statistically significant difference in the T-SPOT positivity rate between Group A and Group C and no statistically significant difference in the mean T-SPOT positivity rate between the two groups. There was no difference in the diagnostic sensitivity of the T-SPOT test in COPD patients compared with that in the normal population. In contrast, it can also be inferred that although COPD patients have decreased numbers of NK cells and CD4^+^ T lymphocytes, as well as reduced cellular immune function, these factors do not affect the rate of positive T-SPOT test.

Table 6 shows a significant difference in the T-SPOT positivity detection rates between Group A and Group B. The difference in mean T-SPOT values between Group A and Group B was statistically significant. It can be speculated that the T-SPOT is highly sensitive in diagnosing active pulmonary tuberculosis in the COPD population, and a higher mean positive detection rate of the T-SPOT indicates a greater possibility of active tuberculosis.

However, one of our main limitations of this study is the lack of data on participants’ smoking history, the duration until their COPD diagnosis, and their history of tuberculosis diagnosis. Also, for some participants, we do not have information about their diagnosis, history of their number of hospitalization due to their condition and their length of hospital stay.

In conclusion, in patients with COPD complicated with active pulmonary tuberculosis, the diagnostic sensitivity of the interferon-γ release assay was higher than that of sputum smear microscopy, PCR detection of *mycobacterium tuberculosis* in sputum specimen, and detection of anti-tuberculosis antibodies. COPD did not affect the frequency of T-SPOT cells; the greater the T-SPOT-positive detection rate was, the greater the likelihood of active TB. For patients who are T-SPOT positive but clinically considered to have inactive tuberculosis, regular follow-ups should be performed to observe changes in the patient’s condition [25].

## Data Availability

All materials and data are available at corresponding author.

## Abbreviations

COPD: Chronic obstructive pulmonary disorder
ELISPOT: Enzyme-linked immunospot assay
GOLD: Global Initiative for Chronic Obstructive Pulmonary Disease
HIV: Human immunodeficiency virus
NK: Natural killer
PCR: Polymerase chain reaction
PPD: Purified protein derivative of tuberculin
TB: Tuberculosis
T-SPOT: T-cell spot
WHO: World Health Organization
